# Lifetime Major Discrimination Experiences Moderate the Impact of Depressive Symptoms on Chronic Conditions among Black Americans

**DOI:** 10.3390/healthcare9111528

**Published:** 2021-11-09

**Authors:** Kia Skrine Jeffers, Quenette L. Walton, Millicent N. Robinson, Courtney S. Thomas Tobin

**Affiliations:** 1 School of Nursing, University of California, 700 Tiverton Ave., Box 956918, Los Angeles, CA 90095, USA; 2 Graduate College of Social Work, University of Houston, 3511 Cullen Blvd., Houston, TX 77204-4013, USA; qwalton2@central.uh.edu; 3 Fielding School of Public Health, University of California, 650 Charles E. Young Dr., Los Angeles, CA 90095, USA; millinr2@g.ucla.edu (M.N.R.); courtneysthomas@ucla.edu (C.S.T.T.)

**Keywords:** lifetime major discrimination, depressive symptoms, chronic conditions, Black Americans, structural racism, depression, cardiometabolic conditions, Nashville Stress and Health Study

## Abstract

To clarify the ways in which Black Americans’ experiences of structural racism may influence their mental and physical health in distinct ways, the present study evaluated whether major discrimination moderates the association between depressive symptoms and chronic physical health conditions among this population. *t*-tests and chi-squared tests of significance were used to determine significant differences between women and men. The association between major discrimination and depressive symptoms was examined by assessing mean depressive symptoms scores across levels of major discrimination. ANOVA tests indicated whether there were significant differences in symptom scores across each discrimination category. Additional *t*-tests determined significant gender differences within each level of discrimination. Gender-stratified negative binomial models were used, and odds ratios (ORs) and 95% confidence intervals (CIs) were estimated for the relationship between depressive symptoms, major discrimination, and chronic conditions. Our findings indicated that the association between depressive symptoms and chronic conditions depends on lifetime experiences of major discrimination among Black Americans and varies significantly between women and men. Considering that major discrimination conditioned the depressive symptom-chronic conditions association among our sample, this provides insight into potential pathways for intervention in efforts to offset the detrimental mental and physical consequences of experiencing racism.

## 1. Introduction

Inequities in physical and mental health among racial and ethnic subgroups in the United States have been well-documented. For instance, Black Americans experience chronic cardiometabolic conditions (e.g., diabetes, hypertension, obesity), premature death, and disability at two to four times the rates of their White counterparts [1,2,3]. Black Americans are also more likely to experience comorbidities that are associated with a greater risk of disease progression [4], accelerated physiological aging [5], and reduced quality of life [6]. To inform more effective clinical and community-based interventions needed to improve these health outcomes, there is a pressing need to identify the factors that contribute to heightened risks among Black Americans.

As previous research has demonstrated a significant, and often bidirectional, link between mental and physical health [7,8], efforts to reduce the high rates of chronic physical health conditions (hereafter referred to as “chronic conditions”) among Black Americans may be enhanced by a focus on depressive symptoms among this population. The association between depression and chronic conditions is well-established in the literature. It is estimated that more than one-third of people with chronic illnesses will experience depression in their lifetime [9,10]. Studies have also found an increased prevalence of chronic diseases including asthma, arthritis, cardiovascular disease, cancer, diabetes, hypertension, and obesity, among individuals with depressive disorders [8,11]. Despite relatively low rates of major depression among Black Americans, many studies document elevated levels of depressive symptoms [12,13]. Given that greater depressive symptoms have been linked to worsening physical health and more chronic conditions in the general population, addressing depressive symptoms’ levels may be an important point of intervention to reduce the risk of chronic conditions among Black Americans.

Nevertheless, evidence from prior studies suggest that the association between depressive symptoms and chronic conditions may be distinct among Black Americans. For instance, studies indicate that depressive symptoms are more chronic and physically debilitating among Black relative to White Americans [12,14,15,16]. Other studies showed that Black Americans face more stressors, including lifetime experience of racism and discrimination, which have been linked to both depressive symptoms and chronic conditions [17]. However, in their longitudinal study, Assari, Burgard, and Zivin [16] found a stronger, long-term association between depressive symptoms and chronic medical conditions among White compared to Black Americans. Thus, the significance of depressive symptoms in shaping the risk of chronic conditions among Black Americans remains unclear. Since most studies have utilized comparative approaches to understand the nature of racial disparities in these relationships, additional research is needed to clarify this association and the factors that may contribute to distinct patterns among Black Americans.

As a fundamental determinant of health [3,18,19,20,21], racism may also condition the linkage between depressive symptoms and chronic conditions among Black Americans. Specifically, racism serves as a critical driver of health inequalities by patterning health risks and resources across cultural, institutional, interpersonal, and individual levels. Although most studies have focused on the role of interpersonal forms of racism, such as everyday discrimination, scholars have increasingly recognized the health significance of structural racism [22,23]. While everyday discrimination captures the ongoing slights and hassles that Black Americans face every day (e.g., people treat them with less courtesy or act as if they are afraid of them), major discrimination experiences encompass individuals’ encounters with unfair treatment across numerous social and institutional domains, including housing, employment, and education. As such, major discrimination often represents Black people’s experiences of structural racism and may contribute to distinct health outcomes among this population.

Yet, several limitations of prior research hinder our knowledge of the ways in which major discrimination shapes the onset and progression of mental and physical health co-morbidities among Black Americans. Since many studies only assessed everyday but not major discrimination experiences, our knowledge of racialized, inequitable treatment at a structural level remains limited. In addition, discrimination studies have disproportionately focused on mental health rather than physical health outcomes [24]. This is insufficient given our incomplete understanding of the drivers that undergird the excess rates of poor physical health and disability among Black people. Finally, few studies have considered the indirect pathways through which individuals’ lifetime experiences of major discrimination may modify their health risks. Recent studies suggest that Black Americans’ lifetime stress experiences, including major discrimination, may modify the physical health consequences of depressive symptoms [12]. Thus, it is possible that major discrimination may shape the ways in which depressive symptoms influence the risk of chronic, comorbid mental and physical health conditions among Black Americans.

**Figure 1:**
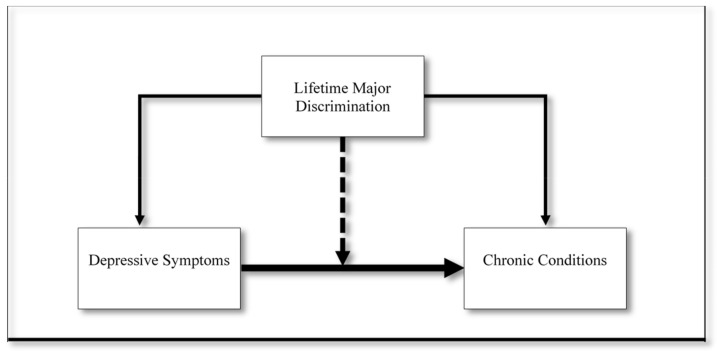
Conceptual model examining the moderating role of lifetime major discrimination on the association between depressive symptoms and chronic conditions among Black Americans.

To clarify the ways in which Black Americans’ experiences of structural racism may influence their mental and physical health in distinct ways, the present study evaluated whether major discrimination moderates the association between depressive symptoms and chronic physical health conditions among this population. Drawing on the conceptual model presented in Figure 1, we addressed three main objectives: (1) assess the association between major discrimination and depressive symptoms among Black women and men; (2) examine the relationships between depressive symptoms, major discrimination, and chronic conditions; and (3) determine whether major discrimination moderates the depressive symptoms–chronic conditions association. Given prior research outlining significant gender differences in the experience of discrimination [25] and depressive symptoms [11], we investigated these relationships separately for Black women and men.

## 2. Materials and Methods

### 2.1. Sample

The Nashville Stress and Health Study (NSAHS) is a population-based sample of Black and White adults aged 21 to 69 from the city of Nashville and surrounding areas within Davidson County, Tennessee. A random sample was obtained using a multistage, stratified sampling approach. Black households were oversampled to achieve a final sample with similar proportions of racial and sex groups, and a sampling weight allowed for the generalizability of sample characteristics to the county population. To account for the rate at which potential study participants were successfully contacted, American Association for Public Opinion Research (AAPOR) rates were used. These AAPOR rates evaluate success across the screening and interviewing phases of data collection (Response Rate 1 = 30.2; Cooperation Rate 1 = 74.2; Refusal Rate 1 = 30.2; Contact Rate 1 = 40.7).

Between 2011 and 2014, 1252 respondents were successfully interviewed about their personal and family backgrounds, stress and coping experiences, health behaviors, and health histories during 3-h computer-assisted interviews with trained study staff of the same race. All participants provided informed consent. Study procedures were approved by the Vanderbilt University Institutional Review Board and have been described in detail elsewhere [26]. The present analyses include data from 627 Black respondents.

### 2.2. Measures

#### 2.2.1. Chronic Conditions

We evaluated the number of self-reported physician-diagnosed chronic physical health conditions as our primary outcome. We assessed nine conditions that were judged to be serious, potentially fatal, and likely to come to the attention of doctors: tuberculosis, diabetes, heart disease, intestinal bleeding, kidney problems, stroke, high cholesterol, liver problems, and pancreatitis. The count was limited to individuals who reported experiencing the condition and responded “yes” to the question “Was this health problem diagnosed by a physician?” [26]. For descriptive purposes, we also considered differences across three categories of chronic conditions: (0) no chronic conditions, (1) one chronic condition, (2) two or more chronic conditions.

#### 2.2.2. Depressive Symptoms

Past-month depressive symptoms were measured with the 20-item Center for Epidemiological Studies Depression (CESD) scale (Cronbach’s alpha = 0.89) [27]. Examples of items included “could not shake off the blues,” “felt depressed,” “sleep was restless,” and “had crying spells.” Response categories ranged from (0) not at all to (3) almost all the time. Items were summed, with higher values indicating higher symptom levels.

#### 2.2.3. Major Discrimination

Exposure to major discrimination was based on a count of 9 lifetime discrimination events from the Major Discrimination Scale [28], which assessed lifetime exposure to unfair treatment across institutional domains such as job promotion, housing, and financial institutions. Respondents were asked whether they experienced each event (e.g., “been unfairly fired or denied a promotion,” “for unfair reasons, not been hired for a job”) and a count variable indicated the number of major discrimination events experienced. In the present analyses, we compared differences across three levels of lifetime exposure: (0) no exposure [0 events], (1) low exposure [1–2 events], and (2) moderate–high exposure [3+ events].

#### 2.2.4. Covariates

Due to their documented association with variations in mental health [12], discrimination [17], and chronic conditions [2], we included age, socioeconomic status, and marital status as covariates. Respondents’ age was measured in years and ranged from 22 to 69 years. Gender distinguished between (0) men, (1) women. Respondents’ current marital status was also assessed and coded as (0) non-married [single, widowed, separated, divorced], (1) married. Finally, respondents’ socioeconomic status (SES) was measured using a standardized index of years of completed education, self-reported annual household income, and level of occupational prestige based on the Nam-Powers-Boyd occupational scores for 2000 [29]. Additional information on the NSAHS coding procedure for occupational prestige can be found elsewhere [30]. In the present study, SES scores were calculated by first standardizing and summing the three dimensions; scores were then divided by the number of dimensions on which data were available [31]. This resulted in an SES score that represents the number of standard deviations above or below the sample’s mean SES, with higher values indicating greater SES. Scores were then categorized by tertiles: (0) low SES, (1) moderate SES, and (2) high SES. By equally weighting education, income, and occupational prestige, this approach captures individuals’ placement within a social hierarchy and provides a comprehensive assessment of SES while reducing data loss on individual indicators [31].

### 2.3. Statistical Analysis

First, we estimated weighted means and frequency distributions for the full sample and by gender. *t*-tests and chi-squared tests of significance were used to determine significant differences among women and men. Next, we examined the association between major discrimination and depressive symptoms by assessing mean depressive symptoms scores across the levels of major discrimination. ANOVA tests indicated whether there were significant differences in symptom scores across each discrimination category. Additional *t*-tests determined significant gender differences within each level of discrimination. We then used gender-stratified negative binomial models, and we estimated odds ratios (ORs) and 95% confidence intervals (CIs) for the relationship between depressive symptoms, major discrimination, and chronic conditions. This approach was utilized because our measure of chronic conditions was a zero-inflated count variable, and the negative binomial distribution allowed us to account for overdispersion. Among both women and men, we examined four models. Model 1 considered the association between depressive symptoms and chronic conditions, while the link between major discrimination and chronic conditions was examined in Model 2. Model 3 examined depressive symptoms and major discrimination simultaneously. Model 4 assessed the interactive association between depressive symptoms and chronic conditions. All models included age, SES, and marital status as covariates. *p*-values under 0.05 were considered statistically significant. All analyses were performed using STATA 15.1.

## 3. Results

### 3.1. Descriptive Statistics

Table 1 summarizes the descriptive statistics for the full sample and by gender. Most individuals did not report any chronic conditions; however, nearly 20% of the sample had at least one condition, while nearly 20% reported two or more conditions. There were no significant gender differences in the number of chronic conditions. The mean depressive symptoms score was 14.33, which was slightly under the cutoff of 16, which indicates clinical significance [27]. There were also significant gender differences, such that Black women had higher symptom levels (m = 16.01, SD = 12.94, *p* < 0.01) relative to Black men (m = 12.27, SD = 11.44, *p* < 0.01). Black women’s symptoms did reach clinical significance. Exposure to lifetime major discrimination was relatively common among Black Americans. For instance, nearly half of the sample (48.95%) reported at least low exposure (1–2 lifetime events) to major discrimination, while 33.47% reported moderate–high exposure (3+ lifetime events). Levels of major discrimination were comparable between women and men.

### 3.2. Association between Major Discrimination and Depressive Symptoms

We examined the association between major discrimination and depressive symptoms among Black women and men in Table 2. Results showed that overall, increased exposure to lifetime major discrimination was associated with significantly higher depressive symptoms (*p* < 0.05). For instance, the mean depressive symptoms score among those with no discrimination exposure was 11.09 (SD = 12.62), relative to depressive symptoms scores of 13.66 (SD = 11.63) and 17.00 (SD = 12.85) among those with low and moderate–high exposure, respectively. While this pattern was observed among both women and men, there was only a significant gender difference in depressive symptoms among individuals who reported no exposure to major discrimination (*p* < 0.05).

### 3.3. Associations between Depressive Symptoms, Major Discrimination, and Chronic Conditions

#### 3.3.1. Black Men

**Figure 2:**
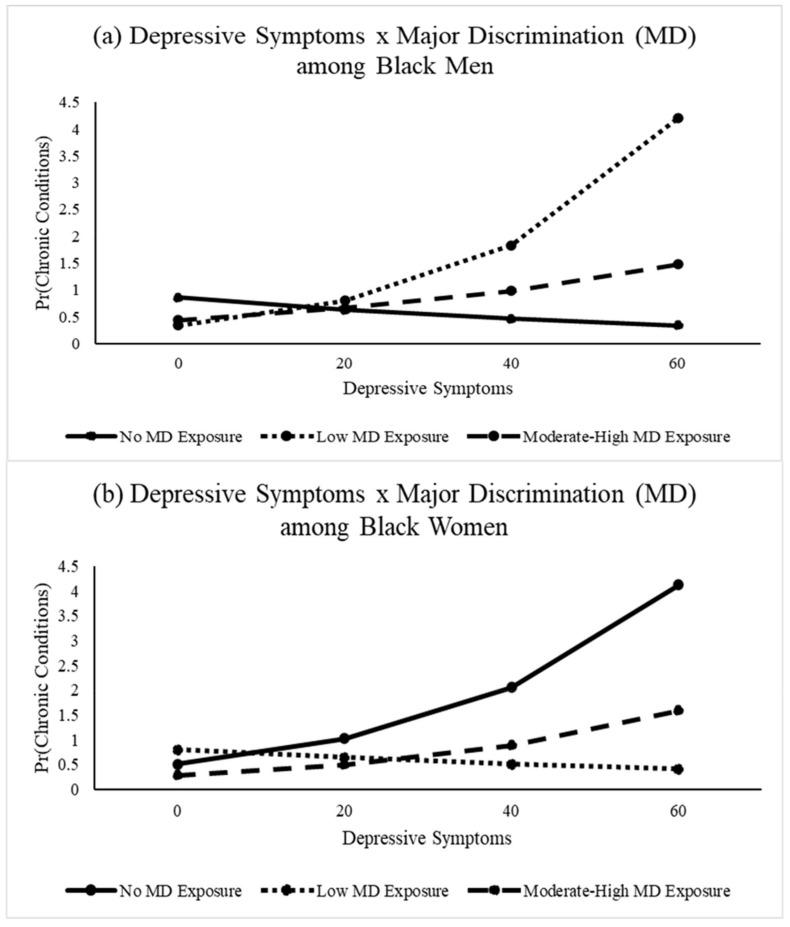
(**a**,**b**) The Association between Depressive Symptoms and Chronic Conditions is Moderated by Lifetime Major Discrimination and Varies Significantly among Black Menand Women. In (**a**), Black men who had experienced low major discrimination had greater odds of chronic conditions. In (**b**), as depressive symptoms increased for Black women, the odds of chronic conditions increased significantly among women with low and moderate–high discrimination exposure. Data: Nashville Stress and Health Study (2011–2014; *N* = 627); age, socioeconomic status, and marital status are included as co0variates.

The relationships between depressive symptoms, major discrimination, and chronic conditions were examined in Table 3. Models 1–3 showed that there were no direct associations between depressive symptoms or major discrimination with chronic conditions among men. However, in Model 4, a significant interaction between depressive symptoms and major discrimination indicated that the relationship between depressive symptoms and chronic conditions among Black men depended on their lifetime exposure to major discrimination. Specifically, the depressive symptoms–chronic conditions association was amplified among Black men who had experienced low levels of major discrimination throughout their lives (OR = 1.06, 95% CI = 1.01–1.12, *p* < 0.05). This distinction is further illustrated in Figure 2a, which shows that the odds of chronic conditions were comparable among Black men with low levels of depressive symptoms. However, as depressive symptoms increased, men who had experienced 1–2 major discrimination events (i.e., low major discrimination) had significantly greater odds of chronic conditions. In contrast, men who reported no discrimination or moderate–high discrimination exposure had similarly low odds of chronic conditions, regardless of their depressive symptomatology.

#### 3.3.2. Black Women

We examined these relationships among women in Table 4. There were no direct associations between depressive symptoms (Model 1) or major discrimination (Model 2) with chronic conditions. However, significant relationships emerged between depressive symptoms and major discrimination, which were considered simultaneously in Model 3. Results showed that higher levels of depressive symptoms were associated with significantly higher odds of chronic conditions (OR = 1.02, 95% CI = 1.01–1.03, *p* < 0.05). Despite a seemingly small effect size, this finding indicates that there is a steady and significant increase in the number of chronic conditions with an increase in depressive symptoms among Black women, such that women with average and high depressive symptoms scores experienced a 32% and 94% increase in the odds of chronic conditions, respectively. Interestingly, women who reported moderate–high exposure to major discrimination had 49% lower odds of chronic conditions once we accounted for differences in depressive symptoms (OR = 0.51, 95% CI = 0.28–0.95, *p* < 0.05).

The emergence of these significant relationships points to a suppression effect, such that failure to account for differences in one of these factors would obscure the significance of the other. This indicates that if discrimination exposure was the same among Black women, then depressive symptoms would have an even greater influence on their chronic conditions. Likewise, if depressive symptom scores were consistent among Black women, then those who faced moderate–high discrimination would have an even greater physical health advantage (i.e., lower odds of chronic conditions) compared to women who reported no discrimination exposure.

Moreover, findings revealed a significant interaction between depressive symptoms and major discrimination, such that the depressive symptoms–chronic conditions association diminished among women with low discrimination exposure relative to those with no discrimination exposure (b = 0.96, 95% CI = 0.92, 0.99, *p* < 0.01). These patterns are further reflected in Figure 2b, which shows comparable odds of chronic conditions among Black women with low depressive symptom levels. Nevertheless, as symptoms increased, the odds of chronic conditions increased significantly among women with low and moderate–high discrimination exposure. There was not a significant association between depressive symptoms and chronic conditions among Black women who reported no exposure to major discrimination.

## 4. Discussion

### 4.1. Key Findings

There is a critical need to improve chronic health conditions among Black Americans. Prior research suggested that depressive symptoms may contribute to this population’s health risks. However, given our limited understanding of the mechanisms linking depressive symptoms to chronic conditions, and recent studies recognizing that Black Americans’ stress experiences may condition the significance of depressive symptoms, we sought to clarify the ways in which Black Americans’ experiences of structural racism may influence their mental and physical health in distinct ways. The present study evaluated whether major discrimination moderates the association between depressive symptoms and chronic conditions among this population. Drawing on the conceptual model presented in Figure 1, we addressed three main objectives: (1) assess the association between major discrimination and depressive symptoms among Black women and men; (2) examine the relationships between depressive symptoms, major discrimination, and chronic conditions; and (3) determine whether major discrimination moderates the depressive symptoms–chronic conditions association. Overall, our findings indicated that the association between depressive symptoms and chronic conditions depends on lifetime experiences of major discrimination among Black Americans and varies significantly between Black women and men.

The present study documents significant associations between major discrimination and depressive symptoms. Specifically, we found that greater exposure to major discrimination events was linked to higher levels of depressive symptoms among Black Americans. Yet, interesting gender differences emerged. While symptom scores were consistently higher among women, there was only a significant gender difference in depressive symptoms among those who reported no exposure to major discrimination. That is, Black women and men who experienced major discrimination tended to experience similar levels of depressive symptoms, while depressive symptoms varied significantly between women and men who experienced no major discrimination. This suggests that experiences of major discrimination may similarly contribute to mental health risk among women and men.

These findings are consistent with previous findings [32,33], which suggest that exposure to major discrimination contributes to higher levels of depressive symptoms among older Black Americans. Nevertheless, additional research is needed to evaluate these patterns across the life course.

Most notably, the findings of the present study demonstrate that the association between depressive symptoms and chronic conditions is distinct between Black men and women. Among men, there were no direct links between depressive symptoms or major discrimination with chronic conditions. Nevertheless, significant interactions revealed that the depressive symptoms–chronic conditions association depends on lifetime experiences of major discrimination among both women and men. Depressive symptoms scores were unrelated to chronic conditions for most men. Yet, increased symptom levels were linked to greater odds of chronic conditions for men with low discrimination exposure (i.e., 1–2 lifetime discrimination events). In particular, elevated symptom levels (i.e., scores of 40+) were linked to heightened odds of chronic conditions among these individuals.

Given that this pattern was only observed among men with low major discrimination exposure, it is possible that men who reported no lifetime discrimination exposure or those who reported moderate–high exposure experience adverse physiological responses (such as elevated blood pressure levels) due to distinctions in the ways in which they may perceive and internalize discrimination events [34,35]. For instance, some suggest that reports of “no discrimination” among Black Americans may actually reflect experiences of “internalized oppression,” which occurs when Black Americans deny or internalize discrimination and consider such experiences as evidence of their own deficiencies rather than systemic inequalities or the biases of others [35]. As such, when faced with unfair treatment, these individuals might deny that such experiences constitute discrimination. This is consistent with findings from the present analyses, which show that Black men who reported no discrimination exposure experienced lower depressive symptoms than those who reported more frequent discrimination exposure. Although whether these patterns arise due to the denial or internalization of discrimination is beyond the scope of the current investigation, our findings that depressive symptoms are unrelated to chronic conditions among these individuals do raise this possibility. While prior studies suggest this internalization may contribute to heightened physiological activity [35], if these individuals do not perceive racial discrimination, it is possible that this denial provides some psychological protection, as reflected in their lower depressive symptom scores and its limited impact on chronic conditions.

In contrast, other studies suggest that those with more central Black identities, who are more likely to perceive more frequent discrimination, may be less vulnerable to the negative effects of discrimination [36]. Thus, while Black men in our study who reported more frequent exposure to discrimination throughout the life course had higher levels of depressive symptoms, the nonsignificant influence of this symptomatology on chronic conditions may arise from a more central Black identity. Future studies evaluating these relationships should evaluate the role of racial identity and other psychosocial processes that shape the ways in which Black men perceive and experience major discrimination to clarify the conditional links between depressive symptoms and chronic conditions among this population.

A different pattern emerged among Black women. Specifically, we observed that depressive symptoms were associated with greater odds of chronic conditions among women who experienced low or moderate–high exposure to major discrimination. These findings suggest that depressive symptoms shape chronic condition risk among Black women who face greater exposure to major discrimination. As we noted previously, it is possible that the nonsignificant link between depressive symptoms and chronic conditions among women with no discrimination exposure may arise from differences in the ways in which these women perceive discrimination. However, the heightened physical health risk associated with depressive symptoms among Black women who have experienced major discrimination throughout their lives might be due to the ways in which women respond to stressful events vary by race, class, and gender and highlights differences in exposure and reactions to stressors [37]. Studies also show that Black American women’s increased physical health risk might be due to their unique experiences of racism and sexism [38,39].

### 4.2. Limitations

The findings of this study should be considered within the context of several limitations. For instance, due to our use of a regional sample from Nashville, Tennessee, we are unable to generalize our findings to broader populations. Relatedly, the analyses presented here were cross-sectional. As such, we are unable to draw conclusions regarding temporal ordering and causality. Given the historical and contemporary experiences of racism and the distinct culture of race relations within many southern U.S. cities, additional research using a nationally representative sample as well as longitudinal data is needed to clarify the extent to which these patterns apply to other contexts and populations. Nevertheless, our results provide an important first step toward understanding how major discrimination impacts the linkages between depressive symptoms and chronic conditions among a socioeconomically diverse sample of Black American adults. Another potential limitation of the current study is our utilization of a measure of self-reported diagnosed chronic conditions. Although prior research has used similar approaches [30], scholars have increasingly recognized the value of evaluating biological indicators of health (e.g., allostatic load) that may provide more insight into the physiological processes underlying disparities [40]. Since gender differences in the prevalence of mental health outcomes are well documented, with women reporting more depressive symptoms, studies should also consider the influence of other mental health indicators beyond depressive symptoms (e.g., externalizing disorders) that Black men may disproportionately face [11]. Lastly, the present study examined the links between major discrimination, depressive symptoms, and chronic conditions, we but did not assess the role of other dimensions of structural racism (e.g., residential segregation). Future research should include other indicators that reflect policy- and structural-level challenges that Black Americans face due to racism.

## 5. Conclusions

Overall, our study demonstrated that the impact of depressive symptoms on chronic conditions depends upon prior exposure to major discrimination among Black Americans. These findings align with recent work assessing the association between psychological distress and psychiatric disorders among Black Americans, indicating that this association is conditional upon stress exposure among Black Americans [12]. Furthermore, results from the current study underscore the importance of examining within-group differences, given that the moderating role of major discrimination in the depressive symptoms–chronic condition association differed between women and men. Taken together, the findings in the present study extend and provide insight into paradoxical health patterns among Black Americans by assessing within-group differences.

The present study makes several significant contributions to the literature. We examined the role of major discrimination as a dimension of structural racism that shapes both physical and mental health outcomes. More specifically, our findings underscore that there are distinct pathways [3,19,21,41] through which experiencing racism across the life course shapes mental and physical health among Black populations. As such, our study provides important insight into understanding the role of racism in shaping the link between physical and mental health among Black Americans. Additionally, our within-group approach to evaluating health patterns among Black Americans contributes to a growing body of evidence highlighting key sources of heterogeneity among Black women and men [42].

Given this, there are substantial implications for future research and practice from these findings. Considering that major discrimination conditioned the depressive symptoms–chronic conditions association in our sample, this has provided insight into potential pathways for intervention in efforts to offset the detrimental mental and physical consequences of experiencing racism. Nevertheless, to comprehensively address this issue and eliminate the health burden of unjust and inequitable systems among Black Americans and other disenfranchised communities, it is of utmost importance to focus on and dismantle the root cause of these health trends, namely racism, to enact a significant change both intentionally and collectively.

## Figures and Tables

**Table 1:** Sample Characteristics among Black Men and Women, Nashville Stress and Health Study (2011–2014).

	All		Black Men		Black Women		
	(*N* = 627)		(*n* = 297)		(*n* = 330)		
	M or %	SD	M or %	SD	M or %	SD	*p*-Value
Number of Chronic Conditions [0–5]	0.75	(1.29)	0.71	(1.29)	0.78	(1.30)	*p* = 0.58
No chronic conditions (Ref.)	52.77		54.30		51.51		*p* = 0.88
One chronic condition	27.78		27.41		28.08		
Two or more chronic conditions	19.45		18.29		20.41		
Depressive Symptoms [0–47]	14.33	(12.58)	12.27	(11.44)	16.01	(12.94)	*p* < 0.01
Major Discrimination							
No Exposure (Ref.)	17.58		16.07		18.83		*p* = 0.24
Low Exposure	48.95		52.70		45.87		
Moderate–High Exposure	33.47		31.23		35.30		
Age [22–69]	43.57	(15.23)	43.33	(15.81)	43.77	(14.75)	*p* = 0.72
Socioeconomic Status (SES) ^a^							
Low SES (Ref.)	28.27		27.12		29.20		*p* = 0.40
Moderate SES	34.35		38.99		30.53		
High SES	37.39		33.88		40.27		
Marital Status							
Unmarried (Ref.)	64.71		53.65		73.80		*p* < 0.01
Married	35.29		46.35		26.20		

Notes: Weighted means or percentages are presented; variable ranges included in brackets; Ref. = Reference category; SD = standard deviation; ^a^ standardized.

**Table 2:** Mean Depressive Symptoms by Level of Lifetime Major Discrimination Exposure among Black Men and Women, Nashville Stress and Health Study (2011–2014).

	All		Black Men		Black Women	
	(*N* = 627)		(*n* = 297)		(*n* = 330)	
	Mean	(SD)	Mean	(SD)	Mean	(SD)
No Exposure (*n* = 137)	11.09 ^a^	(12.62)	8.98 ^bc^	(9.37)	12.57 ^bc^	(14.02)
Low Exposure (*n* = 154)	13.66 ^a^	(11.63)	12.46 ^c^	(9.46)	15.00 ^c^	(11.40)
Moderate-High Exposure (*n* = 125)	17.00 ^a^	(12.85)	14.18 ^c^	(12.76)	19.05 ^c^	(12.15)

Note: This table examines the association between depressive symptoms and major discrimination among Black Americans by comparing mean depressive symptoms scores across levels of discrimination exposure (i.e., no, low, vs. moderate-high exposure). The superscripts “a”, “b”, and “c” indicate statistically significant (*p* < 0.05) differences in depressive symptoms, allowing for multiple contrasts within the full sample and separately among Black men and women. First, superscript (a) indicates that among the full sample (i.e., within the “All” column), there are significant differences in depressive symptoms across levels of discrimination exposure. Second, superscript (b) denotes significant gender differences in depressive symptoms scores within each level of discrimination exposure. In other words, it notes whether men and women with similar levels of discrimination exposure (i.e., no, low, vs. moderate-high exposure) have significantly different depressive symptoms scores. Finally, superscript (c) shows whether there are statistically significant differences in depressive symptoms scores within each gender group (i.e., among men only or among women only).

**Table 3:** Chronic Conditions Regressed on Depressive Symptoms and Lifetime Major Discrimination among Black Men, Nashville Stress and Health Study (2011–2014).

	Model 1		Model 2		Model 3		Model 4	
	OR	95% CI	OR	95% CI	OR	95% CI	OR	95% CI
Depressive Symptoms	1.02	(1.00, 1.05)			1.03	(1.00, 1.06)	0.98	(0.95, 1.03)
Major Discrimination								
No Exposure (Ref.)			1.00		1.00		1.00	
Low Exposure			0.80	(0.43, 1.50)	0.71	(0.38, 1.33)	0.40	(0.15, 1.08)
Moderate–High Exposure			0.81	(0.52, 1.26)	0.67	(0.40, 1.12)	0.51	(0.23, 1.14)
Depressive Symptoms x Major Discrimination (MD)								
Depressive Symptoms x No MD Exposure (Ref.)							1.00	
Depressive Symptoms x Low MD Exposure							1.06 *	(1.01, 1.12)
Depressive Symptoms x Moderate-High MD Exposure							1.03	(0.98, 1.10)

* *p* < 0.05 (two-tailed tests); *n* = 297. Notes: Negative binomial regression models are presented; age socioeconomic status, and marital status are included as covariates; OR = odds ratio; CI = confidence interval; Ref. = reference category.

**Table 4:** Chronic Conditions Regressed on Depressive Symptoms and Lifetime Major Discrimination among Black Women, Nashville Stress and Health Study (2011–2014).

	Model 1		Model 2		Model 3		Model 4	
	OR	95% CI	OR	95% CI	OR	95% CI	OR	95% CI
Depressive Symptoms	1.01	(0.00, 1.02)			1.02 *	(1.01, 1.03)	1.04 **	(1.01, 1.06)
Major Discrimination								
No Exposure (Ref.)			1.00		1.00		1.00	
Low Exposure			0.84	(0.59, 1.18)	0.79	(0.55, 1.15)	1.56	(0.82, 2.96)
Moderate–High Exposure			0.61	(0.34, 1.07)	0.51 *	(0.28, 0.95)	0.55	(0.23, 1.31)
Depressive Symptoms x Major Discrimination (MD)								
Depressive Symptoms x No MD Exposure (Ref.)							1.00	
Depressive Symptoms x Low MD Exposure							0.96 **	(0.92, 0.99)
Depressive Symptoms x Moderate–High MD Exposure							0.99	(0.95, 1.05)

* *p* < 0.05; ** *p* < 0.01 (two-tailed tests); *n* = 330. Notes: Negative binomial regression models are presented; age socioeconomic status, and marital status are included as covariates; OR = odds ratio; CI = confidence interval; Ref. = reference category.

## Data Availability

These data are not publicly available.

## References

[1] Kung H.-C., Hoyert D.L., Xu J., Murphy S.L. (2008). Deaths: Final Data for 2005.

[2] Thorpe R.J., Fesahazion R.G., Parker L., Wilder T., Rooks R.N., Bowie J.V., Bell C.N., Szanton S.L., LaVeist T. (2016). Accelerated Health Declines among African Americans in the USA. J. Urban Health.

[3] Williams D.R., Mohammed S.A. (2009). Discrimination and racial disparities in health: Evidence and needed research. J. Behav. Med..

[4] Kaze A.D., Santhanam P., Erqou S., Ahima R.S., Bertoni A., Echouffo-Tcheugui J.B. (2021). Microvascular Disease and Incident Heart Failure Among Individuals with Type 2 Diabetes Mellitus. J. Am. Hear. Assoc..

[5] Lincoln K.D., Nguyen A.W. (2021). Biopsychosocial Risk Profiles among African American and Non-Hispanic White Adults: Findings from the Health and Retirement Study. J. Gerontol. A Biol. Sci. Med. Sci..

[6] Bazargan M., Cobb S., Assari S. (2021). Completion of advance directives among African Americans and Whites adults. Patient Educ. Couns..

[7] Chapman D.P., Perry G.S., Strine T.W. (2005). Peer Reviewed: The Vital Link between Chronic Disease and Depressive Disorders. Prev. Chronic Dis..

[8] Newcomer J. (2007). Metabolic Considerations in Antipsychotic Medications: A Review of Recent Evidence. J. Clin. Psychiatry.

[9] Cleveland Clinic Chronic Illness and Depression: Causes, Symptoms, Treatment. https://my.clevelandclinic.org/health/articles/9288-chronic-illness-and-depression.

[10] National Institute of Mental Health Chronic Illness and Mental Health: Recognizing and Treating Depression. https://www.nimh.nih.gov/health/publications/chronic-illness-mental-health/.

[11] Walton Q.L., Payne J.S. (2016). Social Work in Mental Health Missing the mark: Cultural expressions of depressive symptoms among African-American women and men. Soc. Work. Ment. Health.

[12] Tobin C.S.T. (2021). Distinguishing distress from disorder: Black-white patterns in the determinants of and links between depressive symptoms and major depression. J. Affect. Disord..

[13] Barnes D.M., Bates L.M. (2017). Do racial patterns in psychological distress shed light on the Black–White depression paradox? A systematic review. Soc. Psychiatry Psychiatrc Epidemiol..

[14] González H.M., Tarraf W. (2013). Comorbid cardiovascular disease and major depression among ethnic and racial groups in the United States. Int. Psychogeriatr..

[15] Watkins D.C., Assari S., Johnson-Lawrence V. (2015). Race and Ethnic Group Differences in Comorbid Major Depressive Disorder, Generalized Anxiety Disorder, and Chronic Medical Conditions. J. Racial Ethn. Health Disparities.

[16] Assari S., Burgard S., Zivin K. (2015). Long-Term Reciprocal Associations Between Depressive Symptoms and Number of Chronic Medical Conditions: Longitudinal Support for Black-White Health Paradox. J. Racial Ethn. Health Disparities.

[17] Lewis T., Cogburn C., Williams D.R. (2015). Self-Reported Experiences of Discrimination and Health: Scientific Advances, Ongoing Controversies, and Emerging Issues. Annu. Rev. Clin. Psychol..

[18] Jones C.P. (2000). Levels of racism: A theoretic framework and a gardener’s tale. Am. J. Public Health..

[19] Phelan J.C., Link B.G. (2015). Is Racism a Fundamental Cause of Inequalities in Health?. Annu. Rev. Sociol..

[20] Williams D.R., Collins C. (1995). US Socioeconomic and Racial Differences in Health: Patterns and Explanations. Annu. Rev. Sociol..

[21] Williams D.R., Mohammed S.A. (2013). Racism and health II: A Needed Research Agenda for Effective Interventions. Am. Behav. Sci..

[22] Ford C.L., Airhihenbuwa C.O. (2010). The public health critical race methodology: Praxis for antiracism research. Soc. Sci. Med..

[23] Walsemann K.M., Geronimus A.T., Gee G.C. (2008). Accumulating Disadvantage Over the Life Course. Res. Aging..

[24] Grollman E.A. (2014). Multiple Disadvantaged Statuses and Health: The Role of Multiple Forms of Discrimination. J. Health Soc. Behav..

[25] Harnois C.E., Ifatunji M. (2010). Gendered measures, gendered models: Toward an intersectional analysis of interpersonal racial discrimination. Ethn. Racial Stud..

[26] Brown T.N., Turner R.J., Moore T.R. (2016). The multidimensionality of health: Associations between allostatic load and self-report health measures in a community epidemiologic study. Health Sociol. Rev..

[27] Radloff L.S. (2016). The CES-D Scale: A Self-Report Depression Scale for Research in the General Population. Appl. Psychol. Meas..

[28] Williams D.R., Yu Y., Jackson J.S., Anderson N.B. (1997). Racial differences in physical and mental health. Socio-economic status, stress and discrimination. J. Health Psychol..

[29] Nam C.B., Boyd M. (2004). Occupational Status in 2000, Over a Century of Census-Based Measurement. Popul. Res. Policy Rev..

[30] Turner R.J., Thomas C.S., Brown T.H. (2016). Childhood adversity and adult health: Evaluating intervening mechanisms. Soc. Sci. Med..

[31] Erving C.L., Thomas C.S. (2018). Race, Emotional Reliance, and Mental Health. Soc. Ment. Health.

[32] Wheaton F.V., Thomas C.S., Roman C., Abdou C.M. (2018). Discrimination and Depressive Symptoms Among African American Men Across the Adult Lifecourse. J. Gerontol. B Psychol. Sci. Soc. Sci..

[33] Nadimpalli S.B., James B.D., Yu L., Cothran F., Barnes L.L. (2014). The Association Between Discrimination and Depressive Symptoms Among Older African Americans: The Role of Psychological and Social Factors. Exp. Aging Res..

[34] Krieger N. (1990). Racial and gender discrimination: Risk factors for high blood pressure?. Soc. Sci. Med..

[35] Krieger N., Sidney S. (2011). Racial discrimination and blood pressure: The CARDIA Study of young black and white adults. Am. J. Public Health.

[36] Sellers R.M., Shelton J.N. (2003). The Role of Racial Identity in Perceived Racial Discrimination. J. Pers. Soc. Psychol..

[37] Keith V.M., Lincoln K.D., Taylor R.J., Jackson J.S. (2010). Discriminatory Experiences and Depressive Symptoms among African American Women: Do Skin Tone and Mastery Matter?. Sex Roles.

[38] Collins P.H. (2000). Black Feminist Thought: Knowledge, Consciousness, and the Politics of Empowerment—Patricia Hill Collins.

[39] Settles I.H. (2006). Use of an Intersectional Framework to Understand Black Women’s Racial and Gender Identities. Sex Roles.

[40] Goosby B.J., Cheadle J.E., Mitchell C. (2018). Stress-Related Biosocial Mechanisms of Discrimination and African American Health Inequities. Annu. Rev. Sociol..

[41] Williams D.R., Mohammed S.A. (2013). Racism and Health I: Pathways and Scientific Evidence. Am. Behav. Sci..

[42] Whitfield K.E., Allaire J.C., Belue R., Edwards C.L. (2008). Are Comparisons the Answer to Understanding Behavioral Aspects of Aging in Racial and Ethnic Groups?. J. Gerontol. Ser. B Psychol. Sci. Soc. Sci..

